# Establishment of a *p53* Null Murine Oral Carcinoma Cell Line and the Identification of Genetic Alterations Associated with This Carcinoma

**DOI:** 10.3390/ijms21249354

**Published:** 2020-12-08

**Authors:** Kuo-Wei Chang, Chia-En Lin, Hsi-Feng Tu, Hsin-Yao Chung, Yi-Fen Chen, Shu-Chun Lin

**Affiliations:** 1 Institute of Oral Biology, School of Dentistry, National Yang-Ming University, Taipei 11221, Taiwan; ckcw@ym.edu.tw (K.-W.C.); megamind@gm.ym.edu.tw (C.-E.L.); esther83427@gmail.com (H.-Y.C.); anice720@hotmail.com (Y.-F.C.); 2 Department of Dentistry, School of Dentistry, National Yang-Ming University, Taipei 11221, Taiwan; hsifeng@gmail.com; 3 Department of Stomatology, Taipei Veterans General Hospital, Taipei 11217, Taiwan

**Keywords:** cancer, mouth, mutation, palate, *p53*

## Abstract

Head and neck squamous cell carcinoma (HNSCC), including oral squamous cell carcinoma (OSCC), ranks sixth in cancer incidence worldwide. To generate OSCC cells lines from human or murine tumors, greatly facilitates investigations into OSCC. This study describes the establishing of a mouse palatal carcinoma cell line (designated MPC-1) from a spontaneous tumor present in a heterozygous *p53* gene loss C57BL/6 mouse. A MPC-1-GFP cell subclone was then generated by lentivirus infection resulting in stable expression of green fluorescent protein. Assays indicated that MPC-1 was a *p53* null polygonal cell that was positive for keratinocyte markers; it also expressed vimentin and showed a loss of E-cadherin expression. Despite that MPC-1 having strong proliferation and colony formation capabilities, the potential for anchorage independent growth and tumorigenesis was almost absent. Like other murine MOC-L and MTCQ cell line series we have previously established, MPC-1 also expresses a range of stemness markers, various oncogenic proteins, and a number of immune checkpoint proteins at high levels. However, the synergistic effects of the CDK4/6 inhibitor palbociclib on other therapeutic drugs were not observed with MPC-1. Whole exon sequencing revealed that there were high rates of non-synonymous mutations in MPC-1 affecting various genes, including *Akap9*, *Arap2*, *Cdh11*, *Hjurp*, *Mroh2a*, *Muc4*, *Muc6*, *Sp110*, and *Sp140*, which are similar to that the mutations present in a panel of chemical carcinogenesis-related murine tongue carcinoma cell lines. Analysis has highlighted the dis-regulation of *Akap9*, *Cdh11*, *Muc4*, *Sp110*, and *Sp140* in human HNSCC as indicated by the TCGA and GEO OSCC databases. *Sp140* expression has also been associated with patient survival. This study describes the establishment and characterization of the MPC-1 cell line and this new cell model should help to advance genetic research into oral cancer.

## 1. Introduction

Head and neck squamous cell carcinomas (HNSCCs), which include oral SCC (OSCC), are prevalent malignancies around the world [1,2]. The establishing of cell lines from human tumor tissues helps us to obtain better mechanistic insights and create better therapeutic interventions that can help control tumor growth [3,4]. Although studies on human OSCC cell lines have allowed there to be remarkable research progress and an identification of new interventions, the immune-compromised preclinical platforms developed from these tumorigenic cell lines are not suitable for addressing questions related to the immune responses that target tumor growth [5,6]. Therefore, murine OSCC cell lines have been developed in the past few years in order to generate immune-competent preclinical models that can be used to assess tumor targeting [2,7,8,9].

4-nitroquinoline 1-oxide (4NQO) is a water-soluble carcinogen that also possesses tobacco-mimicking effects; it induces a range of oxidative species and DNA adducts, as well as acts as a mutagen [10]. It has been widely validated as an inducer of oral carcinogenesis in rodent models [6,10,11]. More than ten 4NQO-based murine tongue SCC cell lines, including six from our laboratory, have been generated [2,7,8,9]. Chen et al. [9] established the NHRI-HN cell line series from the murine tongue tumors induced by 4NQO and arecoline. The syngeneic growth of the NHRI-HN1 cell line was found to be suppressed by an immune stimulant. Wang et al. [2] generated the 4MOSC cell line series from the 4NQO-induced tongue SCC in mice. The mutation profiles of these murine OSCC cells, which resemble their human tumor counterparts, were identified. We generated *miR-211* transgenic mice and found that these mice are highly susceptible to 4NQO-induced OSCC [12]. The MOC-L series of OSCC cell lines were established from 4NQO-induced tumors induced in these mice [8]. Among these, MOC-L1 is highly tumorigenic and metastatic. Cisplatin treatment of MOC-L1 drastically reduced the size of isografts of this tumor line and it was possible to identify in this tumor line the presence of changes in expression of various oncogenic miRNAs, including *miR-196b* [8]. We also established the MTCQ cell line series from 4NQO-induced tongue SCC [7]. MTCQ1 was engineered using GFP to produce a MTCQ1-GFP cell subclone that allowed us to explore the cell line’s metastatic potential. MTCQ1-GFP was found to be sensitive to anti-miRNA and anti-PD-L1 regimens during therapeutic tests [7]. The above findings suggest that establishing more new murine cell lines with diverse genomic or etiological backgrounds should help to accelerate investigations into OSCC.

Notably, *p53* plays a number of versatile roles in tumor suppression [13]. More than 60% human HNSCCs have been reported to carry *p53* mutations, particularly those residing in hot spot codons; these mutations seem to be central to the cell acquiring oncogenicity [1,2]. In addition, a total of eight (73%) of murine OSCC cell lines, including 4MOSC1–4, MOC-L1–L3, and MTCQ1, have been reported to carry *p53* mutations [2,7,8]. Studies of tumors developed from *p53* null mice have allowed us to obtain profound molecular insights into malignant transformation [13]. Furthermore, *p53* null cancer cell lines, such as H1299, HCT116, HN8, and PCI-13, have contributed significantly to our knowledge of the differences in cellular responses and gene regulation between cells with a wild type *p53* and cells with a mutant *p53* [14,15,16,17]. Genomic alternations identified in HNSCC by high throughput sequencing approaches have identified a number of promising gene signatures and networks that might be useful to therapeutic targeting [1,18]. We have established a *p53*-deficient OSCC cell line from a spontaneous tumor in a *p53*^+/−^ mouse. Then we identified the genomic mutations present in this cell line and compared them to other murine OSCC cell lines.

## 2. Results

### 2.1. The Establishment of p53 Null MPC-1 the Cell Lines

**Figure 1:**
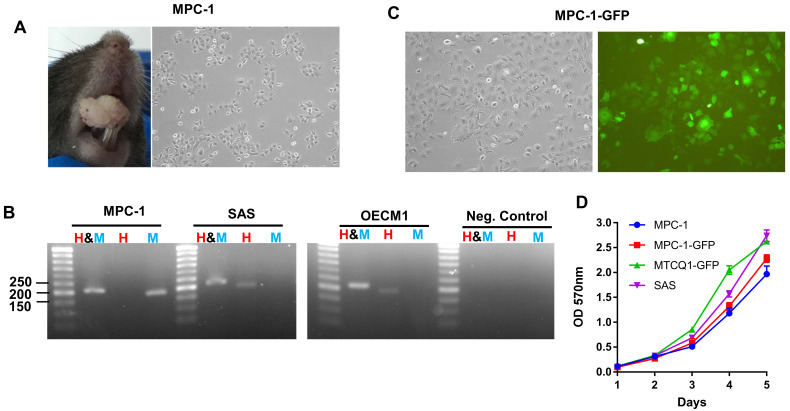
Establishment of the MPC-1 cell line. (**A**) Left, an exophytic tumor growing on the palate of a C57BL/7 mouse. Right, the morphology of the established MPC-1 cell line. Magnification: ×100. (**B**) Genotyping of MPC-1, SAS, and OECM1 cells using PCR to amplify the *PTGER2* gene. The differential amplification and sizes of the PCR products generated by different inputs and primers confirms the mouse origin of the MPC-1 cell line. H & M, both human and mouse; H, human; M, mouse. (**C**) Left, the morphology of the MPC-1-GFP cell subclone. Right, the fluorescence image of MPC-1 cells. Magnification: ×250. (**D**) The growth curves of the MPC-1, MPC-1-GFP, MTCQ1-GFP, and SAS cell lines. Human SAS and OECM1 cell lines, and murine MTCQ1-GFP OSCC cell subclone are served as controls to assay the origin or the grow potential of MPC-1 and MPC-1-GFP.

A 0.5 cm mass occurring on the anterior palate of the oral cavity of a *p53*^+/−^ mouse was noted (Figure 1A, Left). The mouse was sacrificed and the tumor was cultivated to establish a cell line that was designated mouse palatal carcinoma -1(MPC-1). The cells were mainly polygonal or ovoid in shape, and displayed prominent processes (Figure 1A, Right). Genotyping indicated that the cells had originated from a mouse (Figure 1B). A MPC-1-green fluorescence protein (GFP) subclone was then established using lentivirus infection (Figure 1C, Left). The morphology of these cells from this subclone was similar to that of the parental cells, and they also displayed green fluorescence (Figure 1C, Right). The MPC-1 and MPC-1-GFP subclones also shown to have similar growth potential, exhibiting a proliferation capability slightly lower than that of SAS cells and MTCQ1-GFP cells (Figure 1D).

### 2.2. MPC-1 Cell Lacks p53 Protein Expression

**Figure 2:**
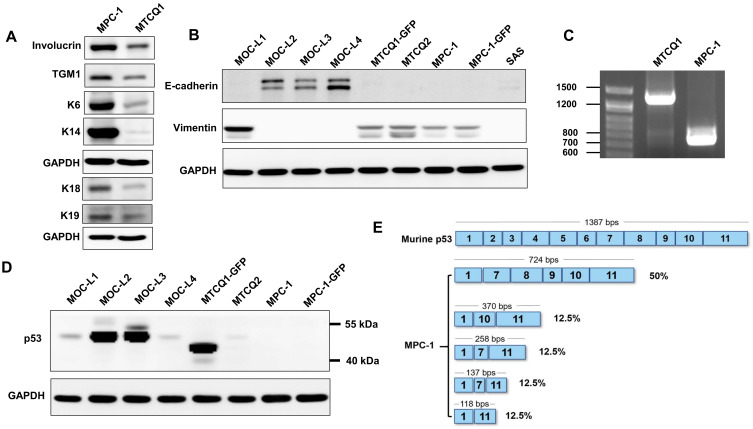
Confirmation of MPC-1 as a *p53* null OSCC cell line. (**A**,**B**,**D**) Western blot analysis. (**A**) Analysis of the keratinocyte markers. The MPC-1 cell line expresses keratinocyte markers much more strongly than the MTCQ1 cell line. (**B**) MPC-1, MOC-L1, MTCQ1, and MTCQ2 are positive for expression of vimentin, but negative for expression of E-cadherin. The other murine OSCC cells express E-cadherin. (**C**) RT-PCR analysis. This shows a ~700 bps *p53* transcript in MPC-1 and a ~1200 bps *p53* transcript in MTCQ1 cell. Note that the bands with a mobility below 600 bps position are not shown in this picture. (**D**) The analysis of *p53* protein in OSCC cells. High *p53* protein expression levels can be seen in the cell lines harboring *p53* mutations. MTCQ1 has a truncated *p53* protein. No *p53* expression can be seen in the MPC-1 cells. (**E**) Sequencing of bacteria colonies carrying RT-PCR products. This reveals the presence of the truncated transcript originally engineered into the knockout mouse (deletion of exon 2–6) in 50% of the white colonies, while four shorter truncated variants make up the remaining 50% colonies.

MPC-1 was found to express various differentiation proteins associated with keratinocytes more abundantly when compared to MTCQ1 cells, these included involucrin, TGM1, and certain keratin variants (Figure 2A). However, the expression of E-cadherin in MPC-1 was completely absent. Instead, vimentin was significantly expressed in MPC-1 (Figure 2B). The E-cadherin and vimentin expression profiles of MPC-1 were similar to other OSCC cell lines and were also in agreement with our previous publications [7,8]; in particular, the MPC-1, MOC-L1, MTCQ1, and MTCQ2 cell lines had very similar E-cadherin and vimentin expression patterns. PCR analysis showed that MPC-1 cells expressed a truncated *p53* transcript that was ~600 bps shorter than the wild type *p53* transcript (Figure 2C). No wild type *p53* transcript could be detected in the MPC-1 cells. Western blot analysis revealed the complete absence of *p53* protein in MPC-1 and MPC-1-GFP (Figure 2D). Sequencing of the *p53* PCR products obtained by RT-PCR from MPC-1 RNA and cloning this into bacteria showed that about 50% of white colonies contained plasmid DNA that included the truncated *p53*; these had a deletion from exon 2 to exon 6. This is consistent with the original knockout mouse design (Figure 2E). The remained 50% of the clones were contained fragments of truncated *p53* that were shorter than the original knockout mouse design.

### 2.3. Phenotypic Analysis of MPC-1

**Figure 3:**
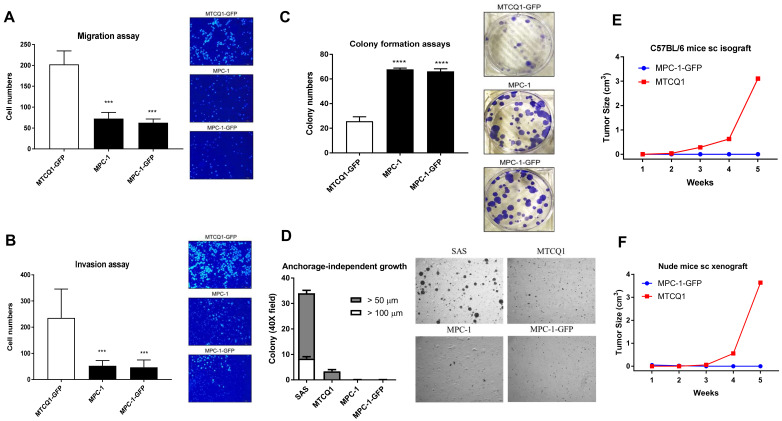
Phenotypic analysis of MPC-1 and MTCQ1. (**A**) Migration. Magnification: ×100. (**B**) Invasion. Magnification: ×100. (**C**) Colony formation. Magnification: ×2. *** *p* < 0.001; **** *p* < 0.0001. (**D**) Anchorage-independent growth. Magnification: ×100. (**A**–**D**), Left panels, quantification. It is performed 48 h (in **A**,**B**) and 10 day (in **C**,**D**) after cell seeding from at least duplicate analysis. Right panels, the representative fields or study sets. The SAS cells in (**D**) act as a side-by-side control. (**E**,**F**) Subcutaneous tumorigenesis in C57BL/6 mice and nude mice, respectively. MPC-1, *n* = 10; MTCQ1 (for side-by-side control), *n* = 1.

Phenotypic analysis showed that MPC-1 had a lower potential for migration and invasion than MTCQ1-GFP (Figure 3A,B). Nevertheless, the colonies formed by MPC-1 were much more abundant and bigger than those formed by the MTCQ1-GFP cell line (Figure 3C). Although MPC-1 showed stronger colony formation ability, the ability of MPC-1 to undergo anchorage-independent growth was almost absent (Figure 3D). The in vitro phenotype of the MPC-1-GFP subclone was almost identical to those of the parent clone. SMPC-1-GFP subcutaneous tumorigenicity in syngeneic mice was absent (Figure 3E). By way of contrast, the isografic growth of MTCQ1 was significant and obvious. To exclude the possibility that immunity was a confounding factor, subcutaneous xenografic growth of MPC-1-GFP in nude mice was also assayed. It was found that the MPC-1-GFP subclone was unable to undergo xenografic growth in nude mice (Figure 3F). The MPC-1-GFP subclone was also found to lack the ability to undergo orthotopic tumor induction in syngeneic mice.

### 2.4. Expression of Stemness Markers, Immune Modulators, and Cell Cycle Regulators in Murine OSCC Cells

**Figure 4:**
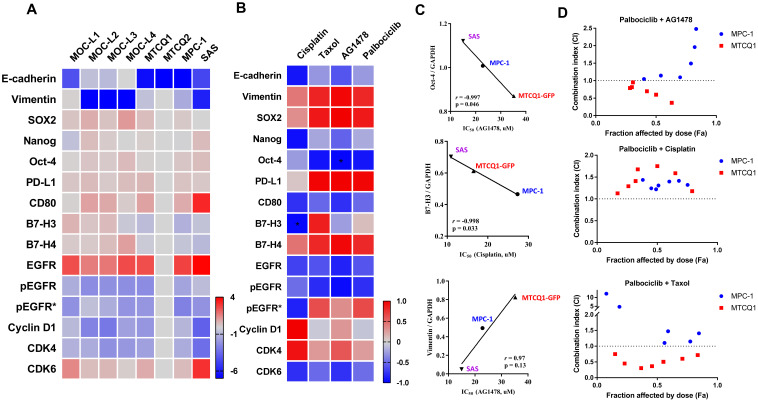
Gene expression profiling and the testing of the effects of various drugs on OSCC cells. (**A**) The heatmap of the normalized protein expression values (log_2_ transformed) for the various cell lines. Right lower, gradient bar. (**B**) The heatmap of correlation values (γ) according to the IC_50_ of the drugs used and protein expression values for MPC-1, MTCQ1, and SAS cells. Right lower, gradient bar. pEGFR*, normalize pEGFR to EGFR. * *p* < 0.05. (**C**) Representative correlation analysis between the IC_50_ of drugs used and protein expression in various cells. (**D**) Analysis of the synergism between palbociclib and AG1478, and between cisplatin and taxol by means of CI for MPC-1 and MTCQ1 cells.

Western blot analysis was performed on MPC-1 and MPC-1-GFP in parallel as well as various other murine OSCC cell lines. The results showed that MPC-1 and MPC-1-GFP expressed stemness markers at a high level (Supplementary Figure S1, Left) as well as some factors that modulate immune escape (Supplementary Figure S1, Right). MPC-1 also expressed EGFR, pEGFR, cyclinD1, CDK4, and CDK6 to a high level (Supplementary Figure S2). The normalized expression levels of the various genes in the various cell lines were transformed into log_2_ values and plotted for comparison (Figure 4A). Except for E-cadherin and vimentin, the expression levels of the various stemness, immune escape, and oncogenic proteins investigated were very consistent across all of the murine OSCC cells. The IC_50_ of the various OSCC cells against AG1478, cisplatin, palbociclib, and taxol was measured by means of dose-response blots. Representative blots are shown in Supplementary Figure S3. The correlation between the IC_50_ for the various drugs and the various genes expressed in MPC-1, MTCQ1-GFP, and SAS were determined and are plotted in Figure 4B. The analysis showed a reverse correlation between cisplatin sensitivity and B7-H3 expression, and between AG1478 sensitivity and Oct-4 expression in these cells (Figure 4C). Although, cisplatin sensitivity is proportional to vimentin expression, no statistically significant correlation was noted. An examination of the combination indices (CI) showed synergism in MTCQ1 cell between palbociclib and taxol, and between palbociclib and AG1478. However, palbociclib did not synergize with any of the tested drugs when inhibition of MPC-1 growth was examined (Figure 4D).

### 2.5. The Gene Mutations Present in Murine OSCC Cells

**Figure 5:**
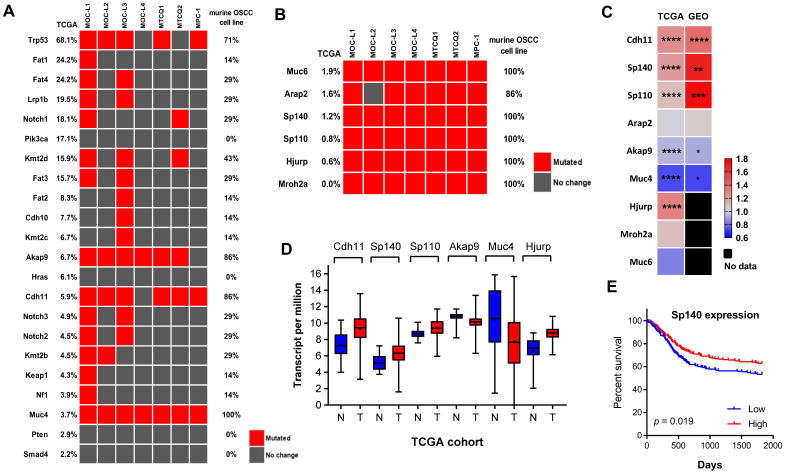
Gene mutations in murine OSCC cells. (**A**,**B**) Heatmap of the mutations present in the various murine OSCC cell lines. (**A**) Analysis according to the order of mutation frequency using the TCGA dataset. (**B**) Analysis according to the presence of mutations in nearly all murine OSCC cells. (**A**,**B**) Left, the mutation frequency based on the TCGA dataset. Right, the mutation frequency in murine OSCC cell lines. A red block indicates the mutated gene. (**C**) Heatmap of gene expression levels in the TCGA and GEO datasets. Right, gradient bar. * *p* < 0.05; ** *p* < 0.01; *** *p* < 0.001; **** *p* < 0.0001. (**D**) Whiskers and a box diagram illustrating the expression levels of genes based on the TCGA human HNSCC database. N, normal tissue, T, HNSCC. (**E**) Kaplan–Meyer survival analysis of the five-year overall survival based on the TCGA human HNSCC database in relation to *Sp140* expression.

Whole genome sequencing revealed various non-synonymous mutations across the panel of murine OSCC cells. Genes with mutation frequencies higher than 2% in TCGA HNSCC tumors are identified in Figure 5A and used as a means of comparing the murine mutation status of the various OSCC cells. The detailed mutation spectra were then integrated into Supplementary Table S3. Overall, *p53* is the gene that has the highest mutation frequency across the murine OSCC cells. Frameshift mutations in exon 2 that result in truncation of the *p53* transcript, were found in MPC-1 and this agrees with the *p53* mRNA expression analysis. The mutation frequencies of *Pik3ca*, *Hras*, *Pten*, and *Smad4* are modest to low in human tumors, and these mutations were also found to be absent in the murine OSCC cells. Despite that the mutation frequency of *Akap9*, *Cdh11*, and *Muc4* being rather low in human tumors, mutations of these genes were found to be very frequent in murine OSCC cells. MOC-L1 had extraordinarily high mutation rates (68%; 15/22) among the listed genes. In addition, 59% (13/22) of the listed genes had a mutation affecting MOC-L3. By way of contrast, MPC-1 and MOC-L4 had the fairly low mutation rates (<14%) for these genes. Mutations affecting the *Fat*, *Notch*, and *Kmt* gene families were detected in 2, 3, and 4 cell lines, respectively.

We also identified ubiquitous mutations in *Muc6*, *Arap2*, *Sp140*, *Sp110*, *Hjurp*, and *Mroh2a* across the murine OSCC cell lines; however, these mutations were assessed as being infrequent or absent in human tumors (Figure 5B; Supplementary Table S4). Interestingly, expression levels of *Sp110*, *Sp140*, *Hjurp*, and *Cdh11* were significantly up-regulated in TCGA tumors, whereas *Akap9* and *Muc4* were significantly down-regulated (Figure 5C,D). This dis-regulation of *Cdh11*, *Sp140*, *Sp110*, *Akap9*, and *Muc4* was also found in the GEO OSCC dataset (Figure 5C, Supplementary Figure S4). Up-regulation of *Sp140* was found to be associated with better patient survival in TCGA tumors (Figure 5E). Overall, MPC-1 and MTCQ1 had rather similar mutated gene profiles.

## 3. Discussion

This study describes the establishing of a new murine OSCC cell line. Murine OSCC cell lines that were established previously were derived mainly from the tongue SCC [2,7,8]. This is the only cell line to be established from a non-tongue oral site. Most of the previously established cell lines are carcinogen-associated tumor cell lines, many created using 4NQO exposure. This is the only cell line that has been developed from a spontaneous tumor without exposure to any carcinogen. Normally, murine OSCC cell lines are either *p53* mutant cell lines or *p53* wild type cell lines [2,7,8,9]. However, MPC-1 is a *p53* null cell line. Since the host is a *p53*^+/−^ mouse, the introduced disruption of the wild type allele in this mouse has resulted in the complete loss of *p53* expression; this loss of expression is likely to be behind the development of this spontaneous tumor. MPC-1 lacks tumorigenic activity despite being eligibility for this in terms of its proliferation and colony formation, which are both potent. However, the anchorage-independent growth potential of MPC-1 is extremely low and thus its lack of tumorigenicity is likely to be due to the difficulties MPC-1 cells have when surviving in adverse microenvironments in vivo. Although murine cell lines possessing tumorigenic eligibility can be used as preclinical models for immune and drug tests, since MPC-1 is *p53* deficient, this cell line is likely to be useful when addressing tumor pathogenesis via restoration of *p53* functionality [15,16].

Although MPC-1 and MTCQ1 display rather similar expression profiles and mutation frequencies for the genes examined in this study, these two cell lines exhibit a much great discrepancy when their phenotypes, drug sensitivities, the effects of drugs in combination, and tumor induction are compared. Inhibition of CDK4/6 in combination with anti-EGFR therapy has been a recent approach used in cancer therapeutics, including the treatment of HNSCC [13,19]. Our preliminary results suggest that palbociclid may work synergistically with taxol or AG1478 to inhibit the growth of MTCQ1 cells. Furthermore, it seems that the *p53* pathway is a crucial component of the effect of palbociclid [19]. Based on the above findings, it is likely that engineered MPC-1 cells that have aberrant *p53* will help to accelerate drug development for HNSCC intervention.

It seems that the differences in E-cadherin and vimentin expression allow us to separate murine OSCC cell lines into two groups. Nevertheless, this study shows that the expression landscape of other oncogenic molecules is generally consistent across the various different cell lines. Apart from mutations affecting *p53*, murine OSCC cell lines also acquire multiple other mutations throughout their genomes. The MOC-L1 and MOC-L3 cell lines carry high mutation burdens, while MOC-L4 and MPC-1 have relatively lower mutation burdens in hotspot genes. The mutation frequencies of *Akap9*, *Cdh11*, and *Muc4* are less than 7% in the TCGA tumor database, whereas their mutation rates are higher than 85% in the murine OSCC cell lines analyzed. Mutations affecting *Arap2*, *Hjurp*, *Mroh2a*, *Muc6*, *Sp110*, and *Sp140* are very rare in the TCGA tumors database, but mutations affecting them are detected in nearly all of the cell lines investigated in this study. The huge size of these genes may partially underlie their high propensity for mutation; nevertheless, these mutations seem to be present overwhelmingly in these cell lines regardless of etiological factors. This suggests that these mutations could be disease-related. Notwithstanding, whether these alterations are simply genetic polymorphisms tightly linked to tumor susceptibility and/or progression, these mutations require more precise investigation. Alternatively, stricter filtration or deeper sequencing are required to increase further the specificity and clarity of the sequencing reads. We have previously identified a 4% and 29% mutation rate of *Smad4* and *Fat1* in HNSCC tissue samples, respectively, which are in agreement with the TCGA tumor database, the results of 4MOSCs analysis and the findings in present study [2,20,21]. Furthermore, this study also identifies a mutation rate of about 60% mutation for *Kmt* gene family members, which is similar to the findings for 4MOSCs [2].

*Akap9*, *Cdh11*, *Muc4*, *Sp110*, and *Sp140* were all found to be dis-regulated in TCGA and GEO datasets. *Akap9* is a kinase-anchoring protein that plays pluripotent roles in coordinating cellular functions and kinase activity. Somatic mutations of *Akap9* are frequent in many cancers, especially in gastrointestinal malignancies [22]. The unique mutation spectrum present in tumor cells and the down-regulation of *Akap9* in tumors needs further study. Multiple missense or indel mutations in *Cdh11* can be found in cells, and *Cdh11* is up-regulated in tumors. However, as a cadherin family member, functional studies suggested that it should have a suppressor role in HNSCC [23]. Both *Muc4* and *Muc6* mucin subtypes are made up of various huge genes, and their mutation profiles are extremely complicated. Dataset analysis has revealed *Muc4* is down-regulation in tumors. However, a significant up-regulation of *Muc4* in HNSCC, which seems to modulate proliferation and senescence, has been reported in a previous study [24]. In addition, silencing of *Muc4* is known to attenuate HNSCC oncogenicity. The mutation spectrum of the nuclear speckled proteins *Sp110* and *Sp140* are complex, and up-regulation of these genes in tumors is intriguing. These molecules mediate transcription regulation and cell apoptosis in immune cells through epigenetic modulation [25]. An in silico module has pinpointed *Sp140* as one of the immune gene hubs that could be involved in HNSCC pathogenesis [26]. The paradoxical association between high *Sp140* expression in tumors and better patient survival is particularly mysterious. The above findings regarding the controversial roles of *Cdh11*, *Muc4*, and *Sp140* in HNSCC pathogenesis, and the interactions between somatic mutation, gene expression, and gene function of these genes needs to be addressed by future investigations.

In summary, this study has established and characterized a murine MPC-1 OSCC cell line. Unlike other murine OSCC cell lines we established from tumors being exposed to 4NQO [7,8], MPC-1 cell line derived for a spontaneous OSCC in *p53*^+/-^ mouse. Only few HNSCC cell lines have been found to be *p53* null [14,17], this new *p53* null cell model will contribute to improving our knowledge of the mechanisms behind HNSCC pathogenesis. In addition, by restoration of wild type or mutant *p53* function, the drug responses of OSCC according to *p53* status can be further elucidated from this cell line. It would also be important to view if the mutant *p53* can augment the aggressiveness of MPC-1. MPC-1 may undertake a unique pathogenic process as it exhibits lesser mutation frequencies, but it also carries mutations commonly present in other murine cell lines. Therefore, continuing in depth genomic analysis of murine OSCC cell lines should also provide further insights into the genetic networks underlying neoplastic progression.

## 4. Materials and Methods

### 4.1. Cell Culture

The heterozygous *p53* knockout mice (Jax stock # 0021; Jackson Lab, Bar Harbor, ME, USA), where there had been a replacement of *p53* exon 2–6 by the neomycin cassette, were a gift of Chun-Min Chen. A spontaneous rapid-growing mass occurred on the anterior palate that bulged outside of the oral cavity of one mouse. This tumor was subjected to cultivation in order to establish a cell line using the protocols we developed previously [7,8]. The tumor cells were designated murine palatal carcinoma -1 (MPC-1). The culture medium used was DMEM (Thermo Scientific, Waltham, MA, USA) containing 10% fetal bovine serum (Thermo Scientific), 1% antibiotic/antimycotic solution (Corning, Corning, NY, USA), and 2 mM l-glutamine (Biological Industries, Grand Island, NY, USA). The cultivation of various other murine cell lines used in this study, including the MOC-L1–L4, MTCQ1, MTCQ2 cell lines, the LL/2 mouse lung cancer cell line, and the SAS and OECM1 human OSCC cell lines followed previously established protocols [4,7,8]. The oncogenic phenotypes, gene expression profiles, and tumorigenic properties of SAS cell line have been rather well characterized [12]. Thus, this human cell lines has been included in the current work and our previous studies to standardize analysis [7,8]. A stable subclone expressing GFP was achieved by sorting of cells after lentiviral infection of MPC-1 [7], and the subclone was designated MPC-1-GFP.

### 4.2. Reagents

The anti-cancer drugs used in this study consisted of EGFR Inhibitor AG1478 (Cat No: ab141438 Abcam, Cambridge, UK), Cisplatin (Cat No: P4394, Sigma-Aldrich, St. Louise, MA, USA), CDK4/6 inhibitor Palbociclib (Cat No: PZ0383; Sigma-Aldrich), and Taxol (Cat No: T7402; Sigma-Aldrich). These were used to test treatment efficacy [27]. All other reagents were purchased from Sigma-Aldrich unless it is specified otherwise.

### 4.3. PCR Analysis

Human and mouse *PTGER2* gene sequences were amplified to confirm the origin of cells (Supplementary Table S1). The murine *p53* transcript spanning exon 1–11 was amplified using the primers described in Supplementary Table S1. The PCR products were cloned into the pHE vector (Biotools, Jupiter, FL, USA) and sequenced [7].

### 4.4. Western Blot Analysis

Western blot analysis followed our previous protocols [8]. The primary antibodies are listed in Supplementary Table S2. Signals were revealed using a luminescent image analyzer (GE Life Sciences, Piscataway, NJ, USA). The signals from the analyzed proteins were normalized against those of GAPDH or another control protein.

### 4.5. Assays for Cell Viability and the Effects of Drugs

Cell viability was analyzed using the MTT assay over 5 days in order to generate growth curves [8]. In addition to the above, cells were seeded and grown for 16–20 h. Next, the cells were treated with the appropriate drugs for 48 or 72 h. Following this, MTT assays were used to measure the viable cell fraction relative to the control. The data were then uploaded to the CompuSyn platform (https://www.combosyn.com/) to obtain CIs [28].

### 4.6. Assays for Migration, Invasion, Colony Formation, and Anchorage-Independent Growth

The migration and invasion assays were performed using Transwell chambers (Merck Millipore, Billerica, MA, USA). For the invasion assay, the Transwell membrane was coated with diluted Matrigel (BD Biosciences, San Jose, CA, USA). After seeding and incubation, the Transwell membranes were fixed and stained using Hoechst 33258. Images of the migrated/invaded cells were captured using a fluorescence microscope [12]. Cells were also seeded at a low density to carry out a colony formation assay. Ten days later, the colonies were fixed and stained with crystal violet. Colonies containing more than 50 cells were counted by microscope [7,8]. Finally, cells were grown in methyl cellulose for 10 days to measure anchorage-independent growth assay; this followed the previously published protocols [12].

### 4.7. Tumor Cell Transplantation

For subcutaneous tumor induction in C57BL/6 mice or nude mice (National Applied Research Laboratory, Taipei, Taiwan), 1 × 10^7^ cells mixed with Matrigel (BD Biosciences) in a total volume of 150 μL were injected into the flanks of the mice. For the orthotopic isograft experiments, 5 × 10^6^ cells mixed with Matrigel in a total volume of 125 μL were injected into a central portion on the tongue of C57BL/6 mice [7,8]. All animal studies were carried out in accordance with the guidelines of the National Yang-Ming University Institutional Animal Care and Use Committee, Approval No: 1070503, Approval Date: 17 May 2018.

### 4.8. Whole Exome Sequencing

Genomic DNA was sent to the company Genomics (New Taipei City, Taiwan) to establish DNA libraries. Sequencing was performed on Illumina HiSeq 4000, using Agilent Sure Select mouse, PE150, 5G data. The reads were aligned (bwa 0.7.17-r1188) to the mm10 mouse reference genome. Duplicated reads were marked by the PICARD (v2.17.2) and base quality recalibration was performed using GATK software (Genome Analysis Toolkit; Broad Institute; Cambridge, MA, USA). The filtered data were merged, re-aligned, and sorted to identify individual non-synonymous mutations.

### 4.9. Statistical Analyses

All data are presented as means ± SE. Mann–Whitney tests, *t*-tests, linear regression, and two-way ANOVA tests were used to compare the differences among the various subsets. Variants from The Cancer Genome Atlas (TCGA, NCI, NIH, Bethesda, MD, USA) and Gene Expression Omnibus (GEO, NCBI, NIH) were retrieved from the relevant websites and analyzed using the UCSC Xena platform (http://xena.ucsc.edu/). Statistical analysis was performed using Prism v7 software (GraphPad, San Diego, CA, USA). A *p* value of less than 0.05 was considered statistically significant. *ns*, not significant; * *p* < 0.05; ** *p* < 0.01; *** *p* < 0.001; **** *p* < 0.0001.

## Supplementary Materials

The following are available online at https://www.mdpi.com/1422-0067/21/24/9354/s1.

## Abbreviations

CI	Combination Index
GFP	Green fluorescence protein
HNSCC	Head and neck squamous cell carcinoma
MPC-1	Mouse palatal carcinoma -1
OSCC	Oral squamous cell carcinoma
4NQO	4-nitroquinoline 1-oxide
