# Supplementary material for: Establishment of a p53 Null Murine Oral Carcinoma Cell Line and the Identification of Genetic Alterations Associated with This Carcinoma
Source: Int J Mol Sci. 2020 Dec 8;21(24):9354. doi: 10.3390/ijms21249354 (PMC7764333; doi:10.3390/ijms21249354)
Supplement: Supplementary file 1 [file ijms-21-09354-s001.pdf]

Supplementary Table S1. Primers used in this study

Gene	Direction	Sequences (5' - 3')	Amplicon (bps)
Human *PTGER2*	Forward	GCTGCTTCTCATTGTCTCGG	189
	Reverse	GCCAGGAGAATGAGGTGGTC	
Mouse *PTGER2*	Forward	CCTGCTGCTTATCGTGGCTG	189
	Reverse	GCCAGGAGAATGAGGTGGTC	
Human and mouse *PTGER2*	Forward	TACCTGCAGCTGTACGCCAC	215
	Reverse	GCCAGGAGAATGAGGTGGTC	
p53 exon 1-11	Forward	CTAGCATTCAGGCCCTCATC	1387
	Reverse	GGCCAGGAACCACTACTCAG	

Supplementary Table S2. Antibodies used in this study

Antibody	MW (kDa)	Host	Dilution	Supplier	Cat. No.
GAPDH	36	mouse	1:10000	Santa Cruz Biotech	sc-32233
E-cadherin	120	mouse	1:1000	BD Biosciences	610182
Vimentin	57	rabbit	1:1000	Cell Signaling	5741
PD-L1	33	rabbit	1:1000	Santa Cruz Biotech	sc-50298
CD80	60	mouse	1:1000	Santa Cruz Biotech	sc-376012
CD86	35	rabbit	1:1000	Abcam	ab112490
CD276	57	rabbit	1:1000	GeneTex	GTX34211
B7-H4	50-75	goat	1:1000	R&D Systems	AF2154
SOX2	35	rabbit	1:1000	Cell Signaling	3579s
Nanog	42	rabbit	1:1000	Cell Signaling	3580s
OCT-4	45	rabbit	1:1000	Cell Signaling	2750s
EGFR	175	rabbit	1:1000	Abcam	ab52894
p-EGFR	175	rabbit	1:1000	Cell Signaling	2234
Cdk4	34	mouse	1:1000	Santa Cruz Biotech	sc-53636
Cdk6	40	mouse	1:1000	Santa Cruz Biotech	sc-53638
Cyclin D1	36	rabbit	1:1000	Cell Signaling	2978
P53	53	mouse	1:1000	Cell Signaling	2524s
Involucrin	120	mouse	1:1000	Sigma-Aldrich	I9018
TGM1	90	goat	1:500	Santa Cruz Biotech	sc-18129
K6	56	mouse	1:1000	MDBio	LVMS766p0
K14	55	mouse	1:1000	Abcam	ab7800
K18	45	mouse	1:1000	Santa Cruz Biotech	sc32329
K19	40	mouse	1:1000	Santa Cruz Biotech	sc376126

Supplementary Table S3: The non-synonymous mutations of genes enlisted in Fig. 5A

Gene name	Cell name	Consequence	Exon (site/total)	cDNA position	Amino acids change	Codon
**p53**	MOC-L1	missense_variant	4/11	522	T/K	aCg/aAg
**p53**	MOC-L1	stop_gained	8/11	944	G/*	Gga/Tga
**p53**	MOC-L2	missense_variant	5/11	623	A/P	Gcc/Ccc
**p53**	MOC-L3	missense_variant	7/11	879	G/E	gGg/gAg
**p53**	MOC-L3	frameshift_variant	11/11	1267	K/X	aaG/aa
**p53**	MTCQ1	missense_variant	6/11	797	V/L	Gtg/Ttg
**p53**	MPC-1	frameshift_variant	2/11	169-170	-/X	-/TC
**p53**	MPC-1	frameshift_variant	2/11	173-174	E/GPC X	gag/gGCCCCTGCag
**p53**	MPC-1	frameshift_variant	2/11	174-175	E/EX	gag/gaGg
**p53**	MPC-1	frameshift_variant	2/11	178-179	/ILPGR GGX	/ATTCTACCG GGTAGGGGA GGCG
**Fat1**	MOC-L1	missense_variant	2/27	2348	V/I	Gtt/Att
**Fat4**	MOC-L1	missense_variant	5/17	6049	S/I	aGc/aTc
**Fat4**	MOC-L1	missense_variant	9/17	9377	D/E	gaC/gaA
**Fat4**	MOC-L1	missense_variant	9/17	11082	S/R	Agc/Cgc
**Fat4**	MOC-L1	missense_variant	9/17	11083	S/N	aGc/aAc
**Fat4**	MOC-L1	missense_variant	16/17	12949	R/L	cGg/cTg
**Fat4**	MOC-L1	missense_variant	17/17	13912	P/L	cCc/cTc
**Fat4**	MOC-L3	missense_variant	9/17	9966	G/R	Ggg/Cgg
**Fat4**	MOC-L3	stop_gained	17/17	13410	E/*	Gag/Tag
**Fat4**	MOC-L3	missense_variant	17/17	14624	R/S	agG/agT
**Lrp1b**	MOC-L1	stop_gained	65/91	11090	E/*	Gag/Tag
**Lrp1b**	MOC-L1	missense_variant	61/91	10613	Q/K	Caa/Aaa
**Lrp1b**	MOC-L1	missense_variant	50/91	8883	G/V	gGa/gTa
**Lrp1b**	MOC-L1	missense_variant	13/91	2847	W/L	tGg/tTg
**Lrp1b**	MOC-L3	missense_variant	75/91	12319	M/I	atG/atC
**Lrp1b**	MOC-L3	stop_gained	43/91	7907	G/*	Gga/Tga
**Lrp1b**	MOC-L3	missense_variant	27/91	5264	D/Y	Gat/Tat
**Notch1**	MOC-L1	missense_variant	6/34	1241	G/V	gGg/gTg
**Notch1**	MOC-L1	missense_variant	4/34	788	C/Y	tGc/tAc
**Notch1**	MTCQ2	missense_variant	12/34	2207	N/S	aAc/aGc
**Kmt2d**	MOC-L1	missense_variant	35/55	9066	K/N	aaG/aaC
**Kmt2d**	MOC-L1	missense_variant	11/55	2810	G/V	gGa/gTa

**Kmt2d**	MOC-L1	missense_variant	11/55	1542	M/I	atG/atA
**Kmt2d**	MOC-L3	missense_variant	25/55	5590	D/N	Gat/Aat
**Kmt2d**	MTCQ2	missense_variant	12/55	3415	L/M	Ctg/Atg
**Kmt2d**	MTCQ2	missense_variant	12/55	3390	R/S	agG/agT
**Kmt2d**	MTCQ2	missense_variant	12/55	3358	P/S	Ccg/Tcg
**Kmt2d**	MTCQ2	missense_variant	12/55	3356	G/V	gGc/gTc
**Kmt2d**	MTCQ2	missense_variant	12/55	3351	D/E	gaT/gaG
**Fat3**	MOC-L1	stop_gained	15/26	10191	C/*	tgC/tgA
**Fat3**	MOC-L1	missense_variant	2/26	816	Q/H	caG/caC
**Fat3**	MOC-L3	missense_variant	22/26	12010	A/S	Gct/Tct
**Fat3**	MOC-L3	missense_variant	22/26	11946	E/D	gaA/gaT
**Fat3**	MOC-L3	missense_variant	18/26	10840	I/F	Atc/Ttc
**Fat3**	MOC-L3	stop_gained	18/26	10839	C/*	tgC/tgA
**Fat2**	MOC-L3	missense_variant	2/24	3212	S/A	Tca/Gca
**Cdh10**	MOC-L3	missense_variant	2/12	596	A/G	gCa/gGa
**Cdh10**	MOC-L3	missense_variant	8/12	1841	S/Y	tCt/tAt
**Cdh10**	MOC-L3	missense_variant	12/12	2647	H/N	Cat/Aat
**Kmt2c**	MOC-L3	missense_variant	40/59	9613	P/T	Cca/Aca
**Kmt2c**	MOC-L3	stop_gained	11/59	1699	E/*	Gaa/Taa
**Akap9**	MOC-L1<br>MOC-L2<br>MOC-L3<br>MOC-L4<br>MTCQ1<br>MTCQ2	missense_variant	39/48	10086	G/A	gGa/gCa
**Cdh11**	MOC-L1	missense_variant	4/13	1504	P/H	cCt/cAt
**Cdh11**	MOC-L2	missense_variant	13/13	3066	D/Y	Gac/Tac
**Cdh11**	MOC-L2	missense_variant	4/13	1388	D/E	gaC/gaA
**Cdh11**	MOC-L3	stop_gained	7/13	1965	G/*	Gga/Tga
**Cdh11**	MOC-L1<br>MOC-L2<br>MOC-L3<br>MTCQ1<br>MPC-1	missense_variant	4/13	1477	V/D	gTt/gAt
**Cdh11**	MOC-L1<br>MOC-L2<br>MOC-L3<br>MTCQ1<br>MPC-1	missense_variant	4/13	1447	R/K	aGa/aAa

**Cdh11**	MOC-L1<br>MOC-L2<br>MOC-L3<br>MTCQ1<br>MPC-1	missense_variant	4/13	1335	I/F	Att/Ttt
**Notch3**	MOC-L1	missense_variant	24/33	3934	R/L	cGc/cTc
**Notch3**	MOC-L3	missense_variant	30/33	5729	Q/H	caG/caT
**Notch2**	MOC-L1	missense_variant	34/34	7306	V/G	gTg/gGg
**Notch2**	MOC-L3	missense_variant	34/34	7247	Q/H	caG/caT
**Kmt2b**	MOC-L1	missense_variant	23/37	4981	G/S	Ggc/Agc
**Kmt2b**	MOC-L1	missense_variant	12/37	3751	D/N	Gac/Aac
**Kmt2b**	MOC-L2	missense_variant	28/37	6048	Q/H	caG/caC
**Keap1**	MOC-L1	missense_variant	4/5	2785	V/L	Gtg/Ttg
**Nf1**	MOC-L1	missense_variant	7/58	901	M/I	atG/atA
**Nf1**	MOC-L1	missense_variant	9/58	1156	L/F	ttG/ttT
**Muc4**	MOC-L1	missense_variant	13/27	8615	N/K	aaC/aaA
**Muc4**	MOC-L1	missense_variant	16/27	9099	V/L	Gtg/Ttg
**Muc4**	MOC-L2	missense_variant	2/27	2730	Y/H	Tat/Cat
**Muc4**	MOC-L2	missense_variant	2/27	2736	S/P	Tcc/Ccc
**Muc4**	MOC-L2	missense_variant	3/27	4257	V/M	Gtg/Atg
**Muc4**	MOC-L2	missense_variant	3/27	4315	S/F	tCc/tTc
**Muc4**	MOC-L3	missense_variant	2/27	616	S/I	aGc/aTc
**Muc4**	MOC-L3	missense_variant	2/27	2238	P/S	Cct/Tct
**Muc4**	MOC-L3	missense_variant	2/27	2248	T/N	aCc/aAc
**Muc4**	MOC-L3	stop_gained	23/27	10203	E/*	Gag/Tag
**Muc4**	MOC-L1<br>MOC-L4	missense_variant	3/27	4455	G/R	Gga/Aga
**Muc4**	MOC-L1<br>MOC-L4	missense_variant	3/27	4459	R/M	aGg/aTg
**Muc4**	MOC-L1<br>MPC-1	missense_variant	2/27	2222	K/N	aaG/aaT
**Muc4**	MOC-L3<br>MTCQ2	missense_variant	3/27	4440	R/G	Aga/Gga
**Muc4**	MOC-L3<br>MPC-1	missense_variant	3/27	4330	S/Y	tCt/tAt
**Muc4**	MOC-L1<br>MOC-L4<br>MPC-1	missense_variant	2/27	2293	H/R	cAt/cGt
**Muc4**	MOC-L1	missense_variant	2/27	2313	T/S	Aca/Tca

	MOC-L4<br>MPC-1					
**Muc4**	MOC-L2<br>MOC-L3<br>MPC-1	missense_variant	3/27	4228	T/S	aCc/aGc
**Muc4**	MOC-L2<br>MOC-L3<br>MPC-1	missense_variant	3/27	4233	P/S	Cct/Tct
**Muc4**	MOC-L2<br>MOC-L3<br>MPC-1	missense_variant	3/27	4243	T/N	aCc/aAc
**Muc4**	MOC-L2<br>MOC-L3<br>MPC-1	missense_variant	3/27	4256	E/D	gaG/gaT
**Muc4**	MOC-L2<br>MOC-L3<br>MPC-1	missense_variant	3/27	4264	T/I	aCa/aTa
**Muc4**	MOC-L2<br>MOC-L3<br>MPC-1	missense_variant	3/27	4284	S/T	Tca/Aca
**Muc4**	MOC-L2<br>MOC-L3<br>MPC-1	missense_variant	3/27	4308	T/S	Aca/Tca
**Muc4**	MOC-L3<br>MTCQ2<br>MPC-1	missense_variant	3/27	4435	D/G	gAc/gGc
**Muc4**	MOC-L1<br>MOC-L3<br>MTCQ2<br>MPC-1	missense_variant	2/27	2196	S/P	Tct/Cct
**Muc4**	MOC-L1<br>MOC-L3<br>MTCQ2<br>MPC-1	missense_variant	2/27	2256	T/P	Aca/Cca
**Muc4**	MOC-L1<br>MOC-L3<br>MTCQ2<br>MPC-1	missense_variant	3/27	4214	S/R	agC/agG
**Muc4**	MOC-L1<br>MOC-L3	missense_variant	3/27	4377	A/P	Gct/Cct

	MTCQ2<br>MPC-1					
**Muc4**	MOC-L1<br>MOC-L3<br>MTCQ2-<br>MPC-1	missense_variant	3/27	4386	T/S	Aca/Tca
**Muc4**	MOC-L1<br>MOC-L3<br>MOC-L4<br>MTCQ2	missense_variant	3/27	4456	G/V	gGa/gTa
**Muc4**	MOC-L2<br>MOC-L4<br>MTCQ1<br>MTCQ2	missense_variant	2/27	2605	S/T	aGc/aCc
**Muc4**	MOC-L2<br>MOC-L4<br>MTCQ1<br>MTCQ2	missense_variant	2/27	2609	N/K	aaT/aaG
**Muc4**	MOC-L2<br>MOC-L4<br>MTCQ1<br>MTCQ2	missense_variant	2/27	2665	R/P	cGt/cCt
**Muc4**	MOC-L2<br>MOC-L4<br>MTCQ1-<br>MTCQ2	missense_variant	2/27	2673	I/F	Att/Ttt
**Muc4**	MOC-L2<br>MTCQ1<br>MTCQ2<br>MPC-1	missense_variant	3/27	4175	Q/H	caA/caT
**Muc4**	MOC-L2<br>MTCQ1<br>MTCQ2<br>MPC-1	missense_variant	3/27	4191	P/S	Cct/Tct
**Muc4**	MOC-L1<br>MOC-L2<br>MOC-L4<br>MTCQ1<br>MPC-1	missense_variant	4/27	6299	E/D	gaA/gaT

**Muc4**	MOC-L1<br>MOC-L2<br>MOC-L3<br>MTCQ2<br>MPC-1	missense_variant	3/27	4414	S/Y	tCc/tAc
**Muc4**	MOC-L1<br>MOC-L4<br>MTCQ1<br>MTCQ2<br>MPC-1	missense_variant	3/27	4299	Q/E	Cag/Gag
**Muc4**	MOC-L1<br>MOC-L3<br>MOC-L4<br>MTCQ2<br>MPC-1	missense_variant	3/27	4407	T/A	Aca/Gca
**Muc4**	MOC-L1<br>MOC-L3<br>MOC-L4<br>MTCQ2<br>MPC-1	missense_variant	3/27	4419	Q/E	Caa/Gaa
**Muc4**	MOC-L1<br>MOC-L3<br>MOC-L4<br>MTCQ2<br>MPC-1	missense_variant	3/27	4423	S/I	aGt/aTt
**Muc4**	MOC-L1<br>MOC-L2<br>MOC-L3<br>MTCQ1<br>MTCQ2-<br>MPC-1	missense_variant	3/27	4171	I/T	aTt/aCt
**Muc4**	MOC-L1<br>MOC-L2<br>MOC-L3<br>MTCQ1<br>MTCQ2<br>MPC-1	missense_variant	3/27	4188	L/F	Ctt/Ttt
**Muc4**	MOC-L1<br>MOC-L2<br>MOC-L3	missense_variant	3/27	4359	P/S	Ccc/Tcc

	MTCQ1<br>MTCQ2<br>MPC-1					
**Muc4**	MOC-L1<br>MOC-L2<br>MOC-L3<br>MTCQ1<br>MTCQ2<br>MPC-1	missense_variant	3/27	4363	I/S	aTc/aGc
**Muc4**	MOC-L1-<br>MOC-L2<br>MOC-L3<br>MTCQ1<br>MTCQ2-<br>MPC-1	missense_variant	3/27	4365	M/L	Atg/Ttg
**Muc4**	MOC-L1<br>MOC-L2<br>MOC-L3<br>MTCQ1<br>MTCQ2<br>MPC-1	missense_variant	3/27	4378	A/G	gCt/gGt
**Muc4**	MOC-L1<br>MOC-L2<br>MOC-L3<br>MTCQ1<br>MTCQ2<br>MPC-1	missense_variant	3/27	4392	H/N	Cac/Aac
**Muc4**	MOC-L1<br>MOC-L2<br>MOC-L3<br>MTCQ1<br>MTCQ2<br>MPC-1	missense_variant	3/27	4401	S/T	Tcc/Acc
**Muc4**	MOC-L1<br>MOC-L3<br>MOC-L4<br>MTCQ1<br>MTCQ2<br>MPC-1	missense_variant	3/27	4324	T/K	aCa/aAa

**Muc4**	MOC-L2<br>MOC-L3<br>MOC-L4<br>MTCQ1<br>MTCQ2<br>MPC-1	frameshift_variant	4/27	6119-6120	-/X	-/GG
**Muc4**	MOC-L2<br>MOC-L3<br>MOC-L4<br>MTCQ1<br>MTCQ2<br>MPC-1	frameshift_variant	4/27	6121	I/X	aTa/aa
**Muc4**	MOC-L2<br>MOC-L3<br>MOC-L4<br>MTCQ1<br>MTCQ2<br>MPC-1	frameshift_variant	4/27	6124	V/X	gTg/gg
**Muc4**	MOC-L2<br>MOC-L3<br>MOC-L4<br>MTCQ1<br>MTCQ2<br>MPC-1	missense_variant	4/27	6159	T/S	Aca/Tca
**Muc4**	MOC-L2<br>MOC-L3<br>MOC-L4<br>MTCQ1<br>MTCQ2<br>MPC-1	missense_variant	4/27	6172	S/L	tCg/tTg

Supplementary Table S4: The non-synonymous mutations of genes enlisted in Fig. 5B

Gene name	Cell name	Consequence	Exon (Site/total)	cDNA position	Amino acids	Codons
**Muc6**	MOC-L1<br>MOC-L3<br>MOC-L4<br>MTCQ1<br>MTCQ2<br>MPC-1	missense_variant	31/33	6254	T/M	aCg/aTg
**Muc6**	MOC-L1	missense_variant	31/33	6149	T/K	aCa/aAa
**Muc6**	MOC-L1<br>MOC-L2<br>MOC-L4<br>MTCQ1<br>MPC-1	missense_variant	31/33	6145	A/T	Gcc/Acc
**Muc6**	MPC-1	frameshift_variant	31/33	5903-5904	P/X	cCC/c
**Muc6**	MPC-1	stop_gained&<br>inframe_insertion	31/33	5901-5902	-/*	-/TAA
**Muc6**	MOC-L1<br>MOC-L3<br>MOC-L4<br>MTCQ1<br>MTCQ2<br>MPC-1	missense_variant	31/33	5353	P/S	Ccc/Tcc
**Muc6**	MOC-L3<br>MOC-L4<br>MTCQ1<br>MTCQ2<br>MPC-1	missense_variant	31/33	5119	A/T	Gcc/Acc
**Muc6**	MOC-L3	frameshift_variant	31/33	4931-4932	G/GX	ggt/ggGGt
**Muc6**	MPC-1	stop_gained&<br>frameshift_variant	31/33	4928-4929	M/I*PLX	atg/atATGAC<br>CACTGGg
**Muc6**	MOC-L3	frameshift_variant	31/33	4926-4927	TM/TX	acCAtg/actg
**Muc6**	MOC-L4	missense_variant	31/33	4927	M/V	Atg/Gtg
**Muc6**	MOC-L1<br>MOC-L2	missense_variant	31/33	4921	S/T	Tcc/Acc
**Muc6**	MOC-L2	missense_variant	31/33	4919	K/M	aAg/aTg
**Muc6**	MOC-L1	missense_variant	30/33	4143	S/R	agC/agG
**Muc6**	MOC-L1	stop_gained	18/33	2364	C/*	tgC/tgA

**Arap2**	MOC-L1<br>MOC-L3<br>MOC-L4<br>MTCQ1<br>MTCQ2<br>MPC-1	missense_variant	2/33	1057	G/W	Ggg/Tgg
**Sp140**	MOC-L1<br>MOC-L2<br>MOC-L4<br>MTCQ2	missense_variant	1/17	461	A/S	Gca/Tca
**Sp140**	MOC-L2<br>MOC-L3<br>MTCQ1<br>MTCQ2<br>MPC-1	stop_gained	2/17	617	R/*	Cga/Tga
**Sp140**	MOC-L4	stop_gained	2/17	623	R/*	Cga/Tga
**Sp140**	MOC-L1<br>MTCQ1	missense_variant	2/17	624	R/Q	cGa/cAa
**Sp140**	MOC-L4<br>MTCQ2<br>MPC-1	missense_variant	3/17	663	E/G	gAa/gGa
**Sp140**	MTCQ1	missense_variant	3/17	742	D/E	gaT/gaA
**Sp140**	MOC-L3<br>MTCQ2	missense_variant	3/17	780	A/V	gCc/gTc
**Sp140**	MOC-L1<br>MOC-L2<br>MOC-L4	missense_variant	6/17	1003	I/M	atA/atG
**Sp140**	MPC-1	missense_variant	8/17	1164	M/T	aTg/aCg
**Sp140**	MOC-L1	missense_variant	9/17	1265	H/D	Cac/Gac
**Sp140**	MOC-L1	missense_variant	9/17	1273	E/D	gaA/gaT
**Sp140**	MOC-L1<br>MOC-L2<br>MOC-L4<br>MTCQ1<br>MTCQ2<br>MPC-1	missense_variant	10/17	1394	R/G	Cgc/Ggc
**Sp140**	MOC-L1<br>MOC-L3<br>MOC-L4<br>MTCQ2	missense_variant	11/17	1460	H/N	Cat/Aat

**Sp140**	MOC-L1<br>MOC-L3<br>MOC-L4<br>MTCQ2<br>MPC-1	missense_variant&<br>splice_region_variant	11/17	1476	E/G	gAg/gGg
**Sp140**	MOC-L2<br>MOC-L3<br>MOC-L4<br>MTCQ1<br>MPC-1	missense_variant	12/17	1509	A/V	gCt/gTt
**Sp140**	MOC-L3	missense_variant	14/17	1662	R/T	aGg/aCg
**Sp140**	MOC-L2	frameshift_variant	15/17	1789-1790	-/X	-/TT
**Sp110**	MOC-L2<br>MOC-L4	missense_variant	12/12	1491	F/C	tTt/tGt
**Sp110**	MOC-L1<br>MOC-L2<br>MOC-L3<br>MOC-L4<br>MTCQ2<br>MPC-1	missense_variant	11/12	1452	E/A	gAg/gCg
**Sp110**	MOC-L1<br>MOC-L2<br>MOC-L3<br>MOC-L4<br>MTCQ2<br>MPC-1	missense_variant	11/12	1439	R/C	Cgt/Tgt
**Sp110**	MOC-L3<br>MPC-1	missense_variant	11/12	1403	G/R	Gga/Aga
**Sp110**	MOC-L2<br>MTCQ2	missense_variant	8/12	1142	Q/K	Cag/Aag
**Sp110**	MOC-L2<br>MOC-L4<br>MPC-1	missense_variant	8/12	1119	Q/L	cAg/cTg
**Sp110**	MOC-L1<br>MOC-L3<br>MOC-L4<br>MTCQ2<br>MPC-1	missense_variant	7/12	945	R/H	cGt/cAt
**Sp110**	MOC-L3<br>MTCQ1	frameshift_variant	6/12	899	E/X	Gaa/aa

	MTCQ2<br>MPC-1					
**Sp110**	MOC-L3<br>MTCQ1<br>MTCQ2<br>MPC-1	frameshift_variant	6/12	894-895	A/AX	gcc/gcAc
**Sp110**	MOC-L2<br>MOC-L4	missense_variant	4/12	728	A/T	Gct/Act
**Hjurp**	MOC-L1<br>MOC-L2<br>MOC-L3<br>MOC-L4<br>MTCQ2<br>MPC-1	missense_variant	8/8	2164	P/L	cCc/cTc
**Hjurp**	MOC-L1<br>MOC-L3<br>MOC-L4<br>MTCQ1<br>MTCQ2<br>MPC-1	missense_variant	8/8	2139	D/N	Gat/Aat
**Hjurp**	MOC-L1<br>MOC-L3<br>MOC-L4<br>MTCQ1<br>MTCQ2-<br>MPC-1	missense_variant	8/8	2134	F/S	tTt/tCt
**Hjurp**	MOC-L1<br>MOC-L3<br>MOC-L4<br>MTCQ1<br>MTCQ2<br>MPC-1	missense_variant	8/8	2070	S/P	Tct/Cct
**Hjurp**	MOC-L1<br>MOC-L3<br>MOC-L4<br>MTCQ1<br>MTCQ2<br>MPC-1	missense_variant	8/8	2069	D/E	gaC/gaG
**Hjurp**	MPC-1	missense_variant	8/8	1858	C/F	tGt/tTt
**Hjurp**	MOC-L1	missense_variant	8/8	1116	E/K	Gag/Aag

**Hjurp**	MOC-L1<br>MOC-L2<br>MOC-L3<br>MTCQ1<br>MTCQ2-<br>MPC-1	missense_variant	8/8	1108	P/H	cCc/cAc
**Hjurp**	MOC-L2<br>MOC-L3<br>MOC-L4<br>MTCQ1<br>MTCQ2<br>MPC-1	missense_variant	8/8	897	D/N	Gat/Aat
**Hjurp**	MOC-L1<br>MOC-L2<br>MOC-L3<br>MTCQ1	missense_variant	3/8	375	Y/N	Tac/Aac
**Hjurp**	MOC-L2<br>MOC-L3<br>MOC-L4<br>MTCQ1<br>MTCQ2<br>MPC-1	splice_region_variant	2/8	326	Q	caG/caA
**Hjurp**	MOC-L2<br>MOC-L3<br>MTCQ1<br>MTCQ2	missense_variant	2/8	301	R/T	aGg/aCg
**Hjurp**	MOC-L1<br>MOC-L2<br>MTCQ2<br>MPC-1	missense_variant	1/8	200	H/Q	caT/caG
**Mroh2a**	MOC-L1<br>MOC-L3<br>MOC-L4<br>MTCQ1<br>MTCQ2<br>MPC-1	missense_variant	3/42	445	A/E	gCg/gAg
**Mroh2a**	MOC-L1<br>MOC-L3<br>MOC-L4<br>MTCQ1	missense_variant	6/42	817	T/M	aCg/aTg

	MTCQ2<br>MPC-1					
**Mroh2a**	MOC-L1<br>MOC-L2<br>MOC-L3<br>MOC-L4	missense_variant	7/42	891	V/M	Gtg/Atg
**Mroh2a**	MOC-L1<br>MOC-L2<br>MOC-L3<br>MTCQ2	missense_variant	7/42	979	R/Q	cGa/cAa
**Mroh2a**	MOC-L1<br>MOC-L2<br>MOC-L4<br>MTCQ2	missense_variant	10/42	1283	Q/H	caG/caT
**Mroh2a**	MOC-L1<br>MOC-L2<br>MOC-L4<br>MTCQ1<br>MTCQ2<br>MPC-1	stop_gained	13/42	1545	R/*	Cga/Tga
**Mroh2a**	MOC-L1<br>MOC-L2<br>MOC-L3<br>MOC-L4<br>MTCQ1<br>MTCQ2	frameshift_variant	14/42	1713-1723	DTVK/X	GATACT<br>GTAAAg/g
**Mroh2a**	MOC-L1<br>MOC-L3<br>MOC-L4<br>MTCQ1<br>MTCQ2	missense_variant	14/42	1746	G/S	Ggc/Agc
**Mroh2a**	MOC-L1<br>MOC-L2<br>MOC-L4<br>MTCQ1<br>MTCQ2<br>MPC-1	missense_variant	15/42	1864	G/V	gGc/gTc
**Mroh2a**	MOC-L1<br>MOC-L2	missense_variant	17/42	1971	G/R	Gga/Aga

	MOC-L3<br>MTCQ1<br>MTCQ2<br>MPC-1					
**Mroh2a**	MOC-L1<br>MOC-L4<br>MTCQ1<br>MTCQ2<br>MPC-1	missense_variant	17/42	1977	G/C	Ggc/Tgc
**Mroh2a**	MOC-L1<br>MOC-L2<br>MOC-L3<br>MTCQ1<br>MTCQ2<br>MPC-1	missense_variant	19/42	2217	I/L	Atc/Ctc
**Mroh2a**	MTCQ1<br>MPC-1	stop_gained	27/42	3088	W/*	tGg/tAg
**Mroh2a**	MOC-L1<br>MOC-L2<br>MOC-L3<br>MTCQ1<br>MPC-1	missense_variant	27/42	3107	M/I	atG/atT
**Mroh2a**	MOC-L1<br>MOC-L2<br>MOC-L4<br>MTCQ1	missense_variant&<br>splice_region_variant	27/42	3157	C/Y	tGt/tAt
**Mroh2a**	MOC-L2<br>MOC-L3<br>MTCQ1<br>MTCQ2<br>MPC-1	missense_variant	29/42	3286	S/N	aGc/aAc
**Mroh2a**	MOC-L3<br>MOC-L4<br>MTCQ1<br>MTCQ2	missense_variant	31/42	3676	T/I	aCa/aTa
**Mroh2a**	MOC-L2<br>MOC-L3<br>MOC-L4<br>MTCQ2<br>MPC-1	missense_variant	32/42	3787	R/L	cGg/cTg

**Mroh2a**	MOC-L1<br>MOC-L3<br>MOC-L4<br>MTCQ1	missense_variant	34/42	4086	L/I	Ctt/Att
**Mroh2a**	MOC-L1<br>MOC-L2<br>MOC-L3<br>MOC-L4<br>MTCQ1<br>MTCQ2	missense_variant	34/42	4176	L/V	Ctt/Gtt
**Mroh2a**	MTCQ1<br>MTCQ2<br>MPC-1	missense_variant	35/42	4272	E/K	Gag/Aag
**Mroh2a**	MOC-L2<br>MOC-L4<br>MTCQ1<br>MTCQ2	missense_variant	35/42	4300	T/M	aCg/aTg
**Mroh2a**	MOC-L1<br>MTCQ1<br>MTCQ2<br>MPC-1	frameshift_variant	35/42	4311-4312	V/VX	gta/gTta
**Mroh2a**	MOC-L1<br>MOC-L2<br>MOC-L3<br>MOC-L4<br>MTCQ1<br>MTCQ2	missense_variant	36/42	4330	P/L	cCa/cTa

Supplementary Figures

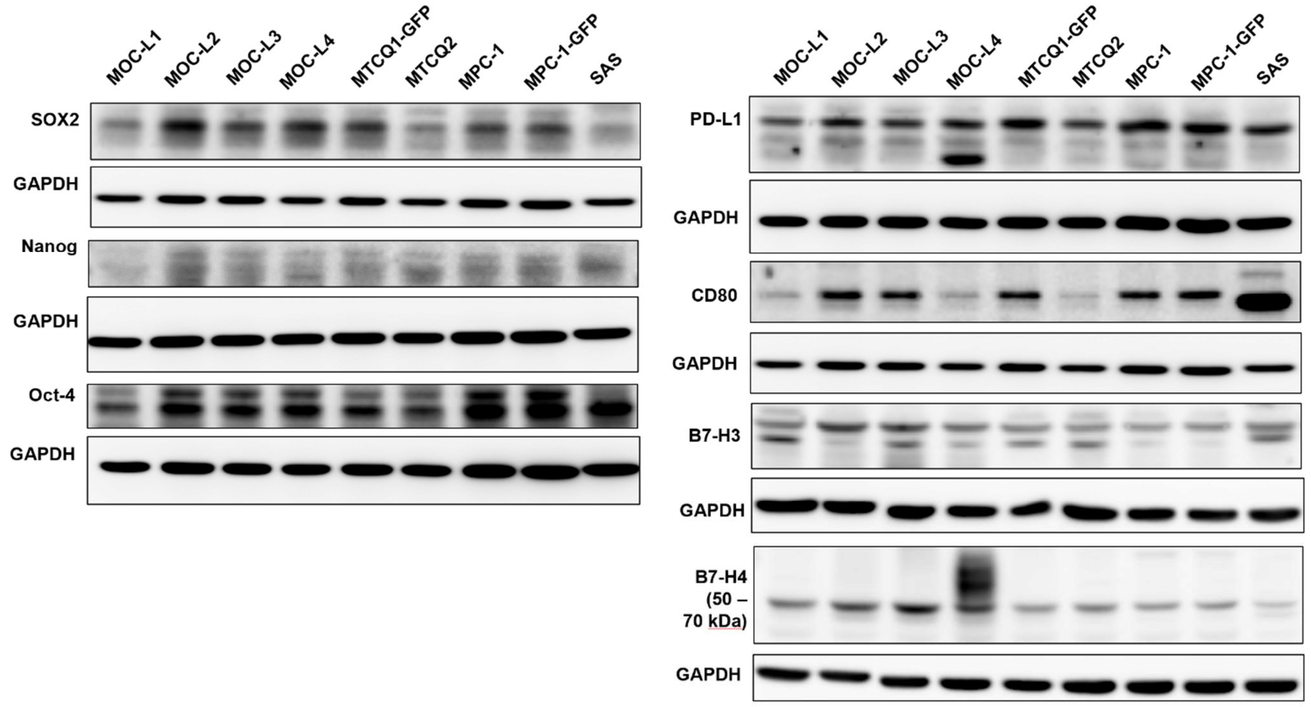

Figure S1. Western blot analysis of OSCC cell lines for various stemness markers (Lt) and immune modulators (Rt).

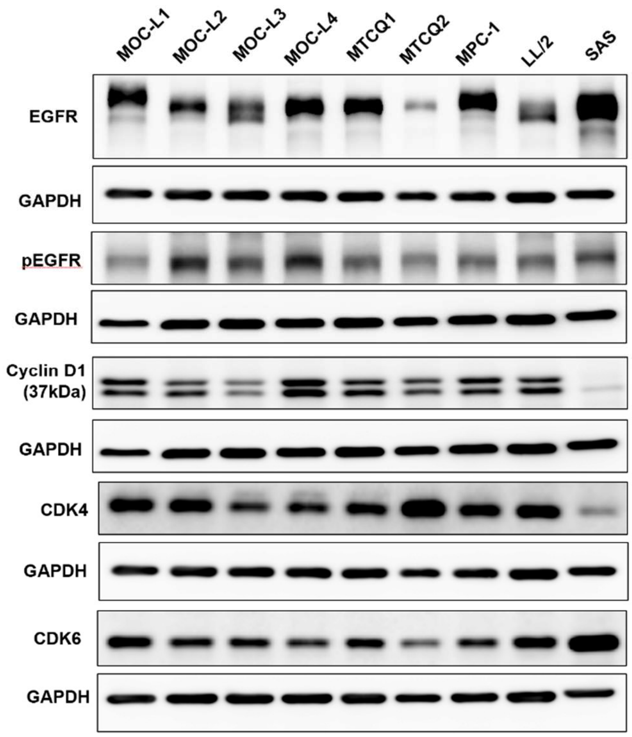

Figure S2. Western blot analysis of OSCC cell lines for various cell proliferation factors.

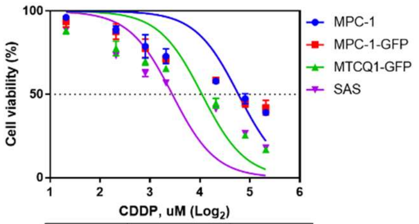

	log IC_{50} (μM)	IC_{50} (μM)
MPC-1	4.774	27.36
MPC-1-GFP	4.771	27.30
MTCQ1-GFP	4.043	16.48
SAS	3.449	10.92

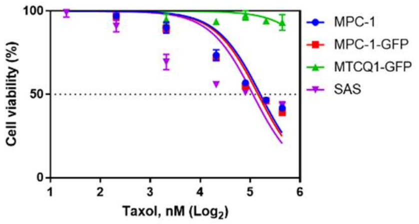

	log IC_{50} (nM)	IC_{50} (nM)
MPC-1	5.206	36.91
MPC-1-GFP	5.161	35.77
MTCQ1-GFP		
SAS	5.057	33.29

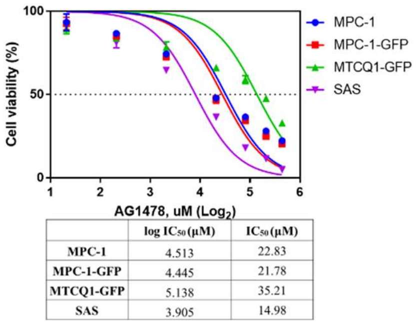

	log IC_{50} (μM)	IC_{50} (μM)
MPC-1	4.513	22.83
MPC-1-GFP	4.445	21.78
MTCQ1-GFP	5.138	35.21
SAS	3.905	14.98

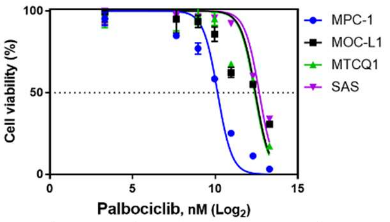

	log IC_{50} (nM)	IC_{50} (nM)
MPC-1	10.15	1136
MOC-L1	12.44	5556
MTCQ1	12.39	5367
SAS	12.67	6517

Figure S3. Representative drug-response curves (upper panels) and the calculated IC_{50} values (lower panels) for the various cell lines and the various drugs.

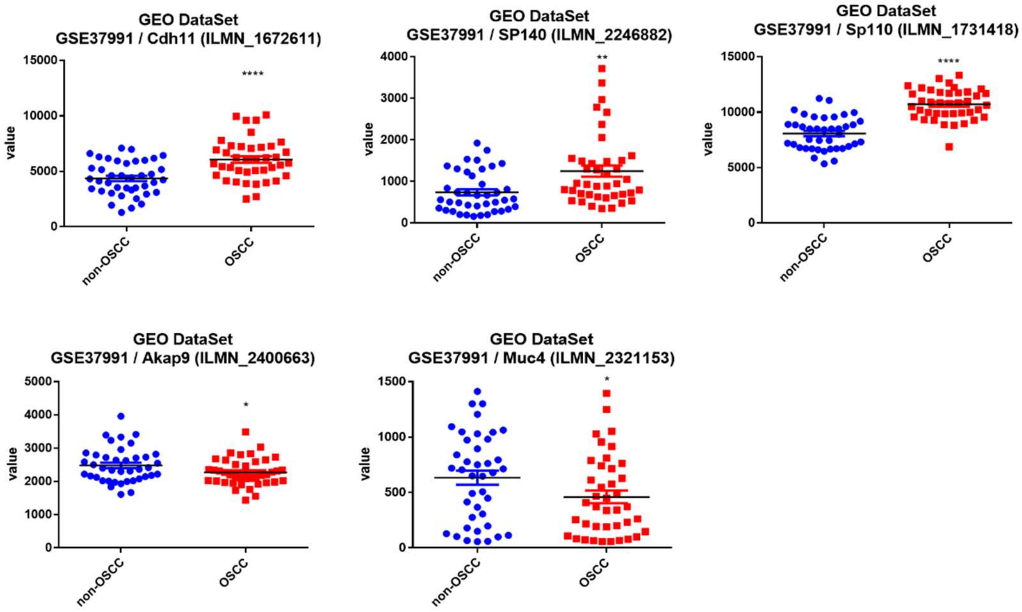

Figure S4. Gene expression status based on the GEO GSE37991 OSCC dataset. This reveals that there is up-regulation of *Cdh11*, *Sp140* and *Sp110*, and down-regulation of *Akap9* and *Muc4* in OSCC relative to the non-OSCC controls. *$p < 0.05$; **, $p < 0.01$; ****, $p < 0.0001$.
